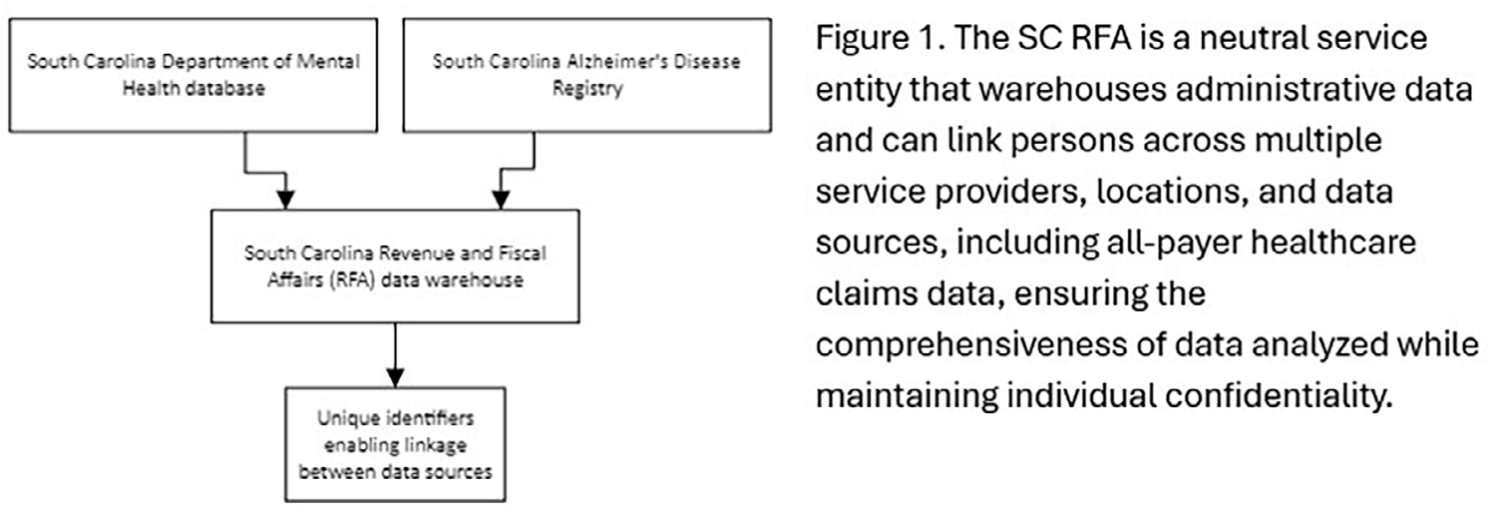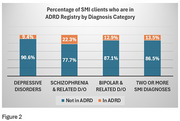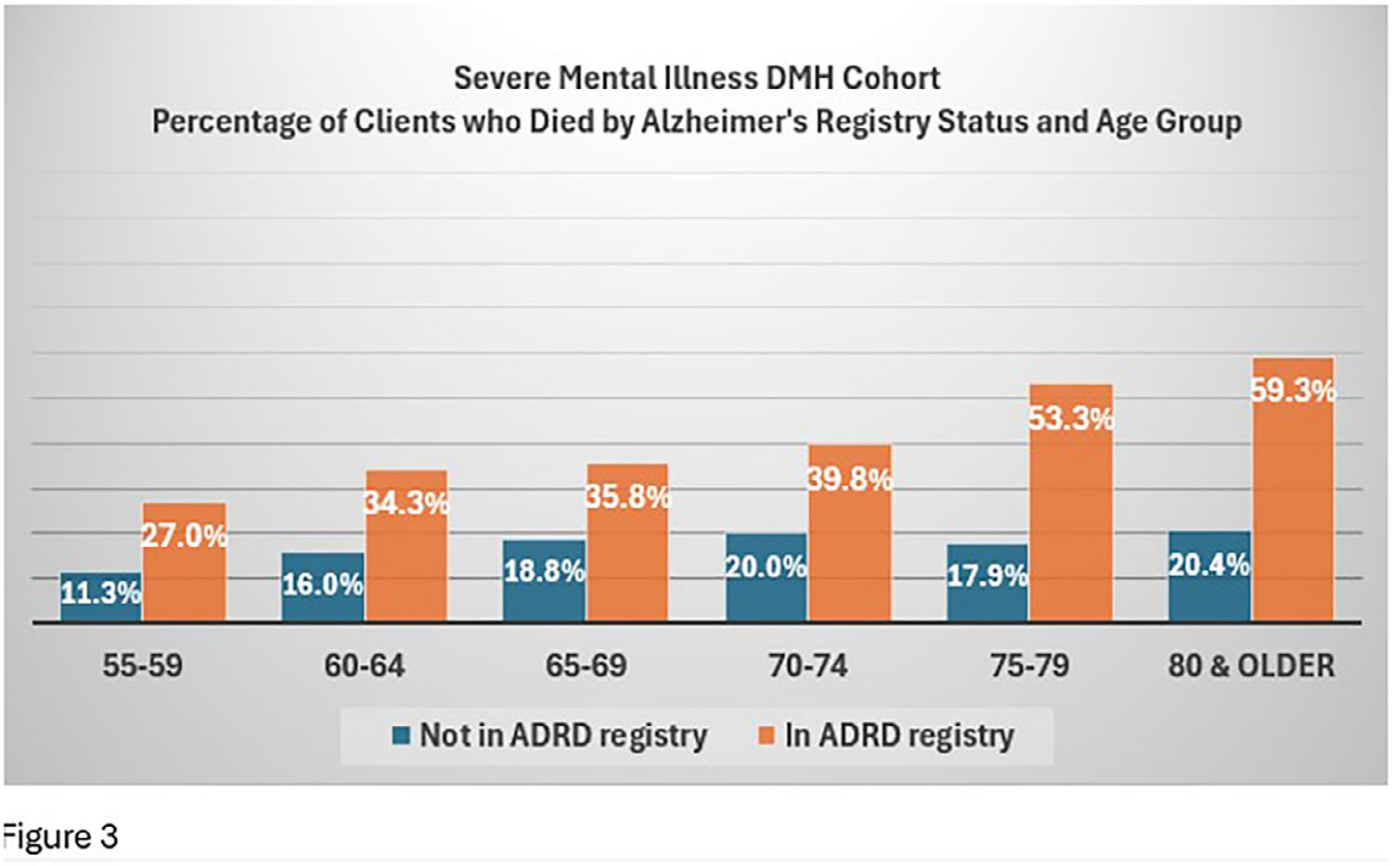# Prevalence of Alzheimer's Disease and Related Dementia in Individuals with Severe Mental Illness: A Retrospective Cohort Study of the South Carolina Alzheimer's Disease Registry

**DOI:** 10.1002/alz70860_099480

**Published:** 2025-12-23

**Authors:** Shilpa Srinivasan, Charlotte Imlach, Julie J Royer, Maggi Miller, Sarah Crawford, Vikram J Kneece

**Affiliations:** ^1^ Prisma Health‐University of South Carolina School of Medicine Columbia, Columbia, SC, USA; ^2^ Prisma Health Columbia SC, Columbia, SC, USA; ^3^ SC Revenue and Fiscal Affairs Office, Columbia, SC, USA; ^4^ University of South Carolina, Columbia, SC, USA

## Abstract

**Background:**

Epidemiological studies suggest individuals with severe mental illness (SMI), including schizophrenia, schizoaffective, and bipolar‐related disorders may be at higher risk for cognitive decline or dementia, but studies on dementia prevalence in this group, particularly in the U.S. public psychiatry sector, are limited. The South Carolina Alzheimer's Disease Registry is the oldest and most comprehensive of three statewide population‐based registries of Alzheimer's Disease and Related Disorders (ADRD) in the U.S. This registry will be utilized to assess the prevalence of dementia in adults with SMI and other associated clinical outcomes.

**Method:**

Characteristics, prevalence of ADRD, and health outcomes of individuals, age 55+ years seen by the South Carolina Department of Mental Health (SCDMH) between fiscal years (FY) 2018‐2022 is analyzed by linking patient data between databases, described in Figure 1. Healthcare outcome is analyzed by linking to patient data from the South Carolina Revenue and Fiscal Affairs (RFA) data warehouse and the Registry. SCDMH IRB approval was obtained. Data in individuals with SMI and ADRD was compared to age/sex/race‐matched persons with SMI without ADRD and analyzed. Results will be stratified or adjusted by diagnosis group to understand the impact of these factors on cognitive comorbidity prevalence, mortality, and health care utilization.

**Result:**

24,291 individuals with SMI received clinical services at SCDMH during the study period. 64.8% of individuals with SMI were female, 58% were White, 37.6% were Black. During the study period, the yearly percentage of individuals with SMI who were reported in the Alzheimer's Registry ranged from 9.7‐17%. The prevalence of AD was higher in individuals with schizophrenia vs. other SMI diagnoses (Figure 2), *p* <0.0001. Mortality rates were higher in individuals with SMI and ADRD vs. SMI only (Figure 3), *p* <0.0001.

**Conclusion:**

ADRD in SMI is prevalent, and associated with increased mortality. ADRD is more prevalent in individuals with schizophrenia than other SMI. Future research on ADRD within SMI populations can inform patient care, service delivery, and long‐term healthcare outcomes.